# Postoperative choroidal vascularity index after the management of macula-off rhegmatogenous retinal detachment

**DOI:** 10.1186/s40942-023-00454-z

**Published:** 2023-03-29

**Authors:** Miguel A. Quiroz-Reyes, Erick A. Quiroz-Gonzalez, Miguel A. Quiroz-Gonzalez, Virgilio Lima-Gomez

**Affiliations:** 1 Oftalmologia Integral ABC, Retina Department, Medical and Surgical Assistance Institution (nonprofit Organization), Affiliated with the Postgraduate Studies Division, National Autonomous University of Mexico, Av. Paseo de las Palmas 735 Suite 303, Lomas de Chapultepec, Mexico City, 11000 Mexico; 2 Retina Department, Oftalmologia Integral ABC, Medical and Surgical Assistance Institution (nonprofit Organization), National Autonomous University of Mexico, Mexico City, Mexico; 3 Institute of Ophthalmology, Fundacion Conde de Valenciana, Medical and Surgical Assistance Institution (nonprofit Organization), Affiliated with the Postgraduate Studies Division, National Autonomous University of Mexico, Av. Paseo, Mexico City, Mexico; 4 Juarez Hospital, Public Assistance Institution (nonprofit Organization), Av. Politecnico Nacional 5160, Colonia Magdalena de las Salinas, Mexico City, 07760 Mexico

**Keywords:** Rhegmatogenous retinal detachment, Buckling procedures, Primary vitrectomy, Choroidal vascularity index

## Abstract

**Background:**

Rhegmatogenous retinal detachment (RRD) is a vision-threatening condition that can be treated with various surgical approaches. The role of scleral buckling remains controversial because of its potential long-term deleterious effects on choroidal vascular perfusion and the limited knowledge of this entity.

**Methods:**

A total of 135 eyes were retrospectively selected, including 115 with surgically resolved RRD and 20 healthy control eyes. Of the surgically treated eyes, 64 underwent vitrectomy alone, while 51 underwent scleral buckling combined with vitrectomy. Best-corrected visual acuity (BCVA) was evaluated along with the choroidal vascularity index (CVI) as a metric for the state of the choroidal vasculature. BCVA was compared before and after surgery, and the postoperative BCVA was analyzed with the CVI using correlation and multivariate regression analyses.

**Results:**

The preoperative BCVA of the RRD eyes was significantly worse than that of the control eyes, and significantly improved after surgery. However, the long-term postsurgical BCVA was still inferior to that of the control eyes. No significant differences in visual function were found between the two surgical groups. The average CVI was 57.35% in the control eyes, 63.76% in the eyes that underwent vitrectomy, and 53.37% in buckled eyes. The differences in CVI were significant among the three groups. Among the surgical patients, negative Pearson’s correlations were found between CVI and postoperative BCVA (expressed in logMAR). A multivariate linear regression model containing four parameters revealed that CVI was the only variable with a significant influence on postoperative BCVA, while the length of time with a detached macula did not have an effect.

**Conclusions:**

RRD surgery drastically restored vision, but the effect of RRD lingered, as postsurgical visual acuity remained inferior to that of the control eyes. The CVI varied between the treatment groups, likely due to both disease pathology and the impact of the surgery. The correlation between CVI and BCVA indicates the important role that the choroidal vasculature plays in visual function.

**Supplementary Information:**

The online version contains supplementary material available at 10.1186/s40942-023-00454-z.

## Key message

1.  RRD is a difficult condition to manage. Although vision is greatly improved with surgery, it is still inferior to that of healthy eyes.
2.  Buckled RRD eyes showed substantial alterations in the choroidal vascularity index relative to non-buckled RRD eyes.
3.  The choroidal vascularity index is associated with postsurgical visual function in eyes with RRD.

**Supplementary Information:**

The online version contains supplementary material available at 10.1186/s40942-023-00454-z.

## Summary statement

To determine the choroidal vascularity index in eyes with successfully operated RRD treated with primary vitrectomy vs. scleral buckling combined with vitrectomy and compare it with that in healthy control eyes.

**Supplementary Information:**

The online version contains supplementary material available at 10.1186/s40942-023-00454-z.

## Background

Rhegmatogenous retinal detachment (RRD) is the most common of the three types of retinal detachment [1]; it is characterized by one or more retinal breaks that allow liquefied vitreous humor to enter the subretinal space, thus separating the neurosensory retina from the underlying retinal pigment epithelium (RPE) [2]. The vitreous liquefaction, the vitreous traction and a retinal break need to be present for RRD formation.

RRD cases can be separated into two groups based on whether the macula is involved or not. Macula-off RRD is characterized by subretinal fluid (SRF) in the subfoveal space, which can lead to permanent photoreceptor damage and visual acuity loss [2]. Macula-off RRD shows better visual acuity restoration when surgery is performed soon after the diagnosis [3, 4].

RRD is a relatively common condition in the general population, with an incidence of approximately 1 in 10,000 people per year [5]. Several risk factors have been associated with the development of RRD, including advanced age, retinal detachment in the fellow eye, myopia, cataract surgery, and trauma [6].

Scleral buckling (SB) is a surgical technique that repairs detachment by reducing the tractional force exerted on the retina by the vitreous body. The procedure has a high reattachment success rate of 85–90% [1], but notable side effects include refraction changes, double vision, and eye movement problems. In recent years, vitrectomy has become a popular alternative method for managing retinal detachment via the removal of detachment-associated vitreous humor. Vitrectomy is preferred in cases with complicating factors at the time of presentation, and in eyes with pseudophakic detachment, multiple or posterior retinal breaks, and vitreous opacities. Additionally, multiple studies have demonstrated that the two procedures can be combined to produce superior results in cases of anterior or inferior tears [7, 8]. These procedures and their combined approach have been explained in detail in recent reviews by Warren et al. [9] and Sultan et al. [5].

While highly effective, scleral buckling involves the permanent insertion of an object around the eye, which may alter the normal physiology of the tissue, including restricting circulation. Indeed, previous studies have shown that scleral buckling is associated with reduced tissue blood velocity in the retina and choroid [10, 11]. The choroid is a highly vascularized tissue that provides an essential blood supply to the RPE and outer retina [12]. A restricted blood supply to the choroid may adversely affect the outer retina, potentially disrupting tissue homeostasis and causing chorioretinal tissue damage.

The choroidal vascularity index (CVI) is a recently introduced quantification parameter that represents the vascular status of the choroid based on optical coherence tomography (OCT) images [13]. CVI was developed as a more precise and robust measurement of choroidal vascularity than previous measurements (such as choroidal vessel diameter and choroidal thickness) which have important limitations [14]. Variations in CVI can be used as an indicator of choroidal vasculature changes, but it cannot capture choriocapillaris flow, an important characteristic of the choroidal vasculature [15].

The CVI has proven to be informative in revealing the status of the choroidal vasculature in a variety of disease and treatment conditions [16–21]. The main objectives of this study were as follows: (1) to compare CVI in eyes with surgically resolved macula-off RRD treated with either primary vitrectomy alone or SB combined with vitrectomy; (2) to contribute to the analysis of submacular CVI as a marker; and (3) to create a multivariate linear model to identify potential correlations among final BCVA, age, CVI, the number of weeks with a detached macula, and the follow-up time after surgery.

## Methods

### Study design

This was a non-randomized review of the medical charts of patients with primary and non-complicated RRD. All patients were treated at the Oftalmologia Integral ABC Institution between January 2015 and May 2021. This study followed the guidelines outlined in the Declaration of Helsinki and was approved by the relevant institutional ethics and teaching committee. The participants provided written informed consent for inclusion in the study. This study involved a retrospective review of medical records, and there was no prospective contact with potential study subjects prior to the acquisition of the requested data. No reference number was provided for the retrospective studies by the institution.

A retrospective analysis of 115 consecutive patients with primary, noncomplicated macula-off RRD who underwent primary pars plana vitrectomy (PPV) or SB combined with vitrectomy was conducted. All patients underwent PPV. Supplementary SB was reserved for pseudophakic eyes with RRD associated with an inferior retinal tear. Twenty healthy eyes as a comparative control group were included in the statistical analysis.

The inclusion criteria were as follows: over 18 years of age, evidence of proliferative vitreoretinopathy (PVR) grade B or less, complete retinal attachment at the last follow-up visit, absence of intraocular silicone oil at the last follow-up visit, at least six months of follow-up, a well-documented structural, functional, and perfusion evaluation during follow-up, and an additional CVI assessment of the macula at the last follow-up visit were included.

The exclusion criteria were as follow: prior complicated surgeries (e.g., vitreoretinal surgery or intravitreal injections), RRD due to open eye injuries, trauma-related RRD, macular hole-related RRD in myopic traction maculopathy, high myopia-related RRD (axial length > 26.5 mm), retinoschisis, and giant tear-related RRD. Eyes with intraocular silicone oil during the final evaluation, PVR (posterior or anterior) with recurrent RRD, active glaucoma, and endophthalmitis were also excluded. In addition, patients who were lost to follow-up were excluded.

#### Examinations

Preoperative evaluations included visual acuity measurement, slit-lamp examination, fundoscopy, indirect ophthalmoscopy, and B-scan ultrasonography (A and B Ultrasound Unit, Quantel Medical, Du Bois Loli, Auvergne, France) as previously described [20]. A Spectral-domain OCT (SD-OCT) (RTVue-XR platform SD-OCT; Optovue, Inc., Fremont, CA, USA) device was used to capture postoperative cross-sectional images of the macular region along the horizontal plane through the foveal center. The following postoperative assessments were statistically analyzed for eyes in the surgical groups: age, number of weeks with a detached macula, follow-up time after surgery, CVI, luminal area (LA), and total choroidal area (TCA).

#### Surgical technique

The patient’s charts were divided into two groups: the SB combined with the vitrectomy group and the vitrectomy group. All surgical procedures were performed by an experienced vitreoretinal surgeon (MAQR). Surgical SB and vitrectomy techniques have been previously described [22]. In brief, the buckling component was placed as an initial step using traditional 504 or 503 360° round Lincoff episcleral sponges (Storz model E-5395-4) around the equator of the eye and fixation with polyester 5 − 0 MERSILENE® Polyester Sutures and double-armed 3/8 circle spatulated needle sutures (ETHICON, Johnson & Johnson, Brunswick, NJ, USA). The vitrectomy technique consisted of a standard 25-gauge 3-port PPV with Landers precorneal lenses and an ocular inverter vitrectomy system with a Zeiss microscope or the Resight-Lens system. In addition to central vitrectomy, we used diluted triamcinolone acetonide adjuvant (Kenalog 40 mg/mL; Bristol-Myers Squibb, New York, NY, USA) to mark the vitreous face and remove it from the retinal surface. Next, retinal reattachment and SRF endodrainage were performed with the aid of heavy perfluorocarbon liquid, followed by argon endolaser-based retinopexy around the retinal tears. Finally, a non-expandable bubble containing a gas mixture of 15% perfluoropropane (C_3_F_8_) was used as a long-lasting tamponade in all the cases [23].

#### CVI measurement technique

The postoperative CVI was measured using enhanced depth imaging of the macula. The OCT-B 9 mm high-definition (HD) images were analyzed using the ImageJ program (version 1.53; NIH, Rasband and contributors, USA, public domain) and changed to an 8-bit format. Subsequently, the brightness was lowered to the minimum threshold and the images were binarized. Next, the area of the subfoveal choroid was measured manually between 750 μm nasal and 750 μm temporal in a horizontal plane from the center of the fovea and vertically from the RPE-Bruch’s membrane complex to the scleral border. Finally, LA was identified by counting the dark pixels, which were divided by the TCA to produce CVI values in the form of a percentage.

### Statistical analysis

All statistical analyses were performed using GraphPad Prism version 9.2.0 software and R environment version 4.1.1, with statistical significance set at p < 0.05. The Kruskal-Wallis test with Benjamini and Hochberg correction was used to test for differences in age, preoperative BCVA, postoperative BCVA, and postoperative CVI between the groups. Fisher’s exact test was used to test for differences in the proportion of sexes among study groups. The two-tailed Mann–‒Whitney U test was used to identify differences in the preoperative characteristics between the two surgical groups. The two-tailed Wilcoxon matched-pairs signed-rank test was used to test for BCVA changes after surgery. Nonparametric tests were used because the normality of distribution tests showed that the data were not normally distributed. A multivariate regression analysis was conducted in the R environment using the lm function.

## Results

### Characteristics of the eyes

The present study included 20 healthy control eyes and 115 eyes with macula-off RRD of Hispanic ethnicity that underwent surgery. Of the surgically treated eyes, 64 underwent vitrectomy alone (collectively named the vitrectomy group) and 51 underwent SB combined with vitrectomy (collectively named the buckling group). Four of the 64 (6.3%) vitrectomy group cases required additional vitrectomy revision surgery, while two of the 51 (3.9%) buckling group cases required additional intervention due to re-detachment.

No significant difference in age was found among the three study groups (*p* = 0.642), whereas significant differences in sex distribution were found (*p* = 0.0010). Both surgical groups had significantly worse preoperative BCVA than the control group (*p* < 0.0001); however, no difference in BCVA was identified between the two surgical groups (*p* = 0.53). Patients in the buckling group had, on average approximately a week less time with a detached macula than those in the vitrectomy group, and this difference was statistically significant (*p* = 0.005). No difference in the postoperative follow-up time was found between the two surgical groups (*p* = 0.912). Patient demographic data and clinical characteristics are summarized in Table 1.

**Table 1:** Patient demographic data and clinical characteristics

Patient Characteristics	Control Group	Vitrectomy Group	Buckling Group	P-value
Number of patients (n)	20	64	51	-
Number of females (n)	16	32	41	0.0010
Mean age	48.65	49.64	47.01	0.642
Mean preoperative BCVA (logMAR)	0.00	1.05	1.02	< 0.0001
Meantime with a detached macula (weeks)	-	4.47	3.41	0.0052
Mean postoperative follow-up (months)	-	26.00	25.71	0.912

BCVA, best-corrected visual acuity; logMAR, logarithm of the minimum angle of resolution.

#### RRD surgical outcomes

In both surgical groups, the postoperative BCVA was significantly better than the preoperative BCVA (*p* < 0.0001) (Table 2). The logarithm of the minimum angle of resolution (logMAR) BCVA improved by an average of -0.72 in the vitrectomy group and − 0.60 in the buckling group. However, the postoperative BCVA remained worse than that of the control eyes in both surgical groups (*p* < 0.0001). No significant difference was found between the two surgical groups in either the preoperative (*p* = 0.459) or postoperative BCVA (*p* = 0.168).

**Table 2:** Preoperative and final postoperative BCVA.

Study groups	Mean presurgical BCVA (logMAR)	Mean final BCVA (logMAR)	Mean change in BCVA (logMAR)	*P*
Vitrectomy	1.05	0.33	-0.72	< 0.0001
Buckling	1.02	0.42	-0.60	< 0.0001

BCVA, best-corrected visual acuity; logMAR, logarithm of the minimum angle of resolution.

#### CVI analysis

**Fig. 1:**
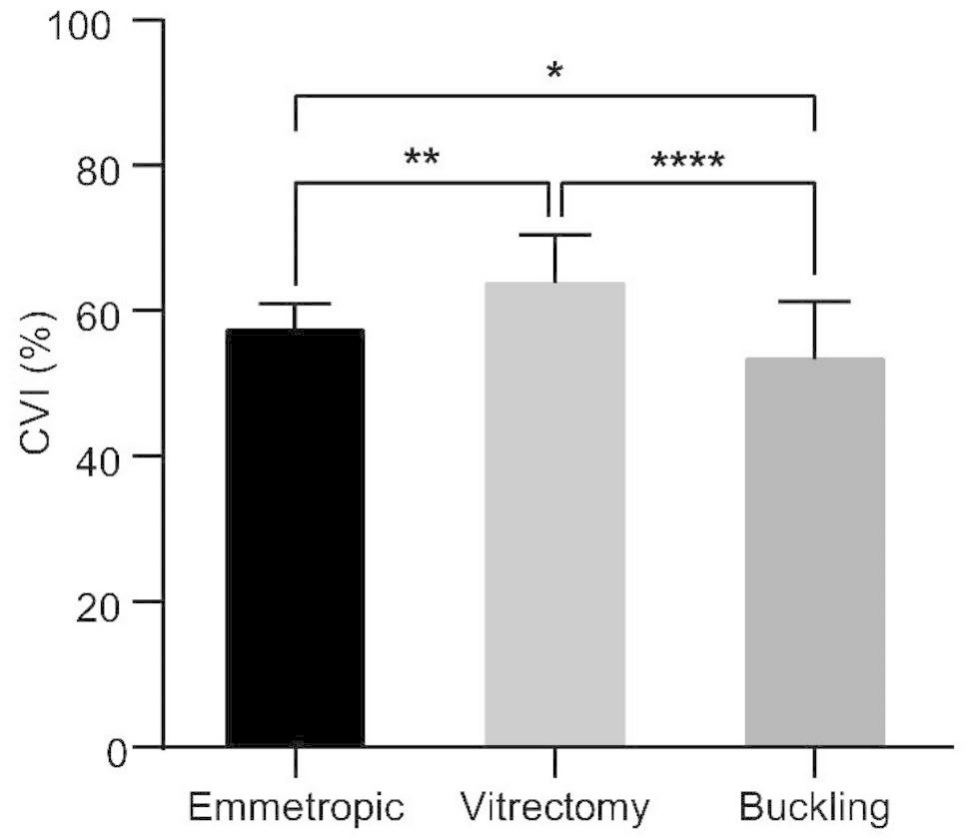
Choroidal vascularity index across study groups Optical coherence tomography images were analyzed using ImageJ to obtain the luminal area (LA) and total choroidal area (TCA). The choroidal vascularity index (CVI) was calculated by dividing LA by TCA and was presented as a percentage. CVI was significantly lower in the buckling group and higher in the vitrectomy group than in the emmetropic group. The mean ± standard deviation is plotted; * *p* ≤ 0.05, ** *p* ≤ 0.01, and **** *p* ≤ 0.0001

The postoperative binarized OCT images were analyzed to obtain LA and TCA values, which were used to calculate the CVI values in the form of percentages **(**Fig. 1**).** The average CVI were 57.35% in the control group, 63.76% in the vitrectomy group, and 53.37% in the buckling group (Table 3). The differences in the CVI between the groups were statistically significant (*p* = 0.002 between the control and vitrectomy group, *p* = 0.049 between the control and buckling group, and *p* < 0.0001 between the vitrectomy and buckling groups).

**Table 3:** Choroidal measurements and calculations

Study groups	LA (mm^2^)	TCA (mm^2^)	CVI (%)	CVI, 95% confidence interval (%)
Control	0.47	0.82	57.35	(55.61, 59.08)
Vitrectomy	0.38	0.60	63.76	(62.07, 65.44)
Buckling	0.31	0.58	53.37	(51.15, 55.59)

LA, luminal area; TCA, total choroidal area; CVI, choroidal vascularity index.

**Fig. 2:**
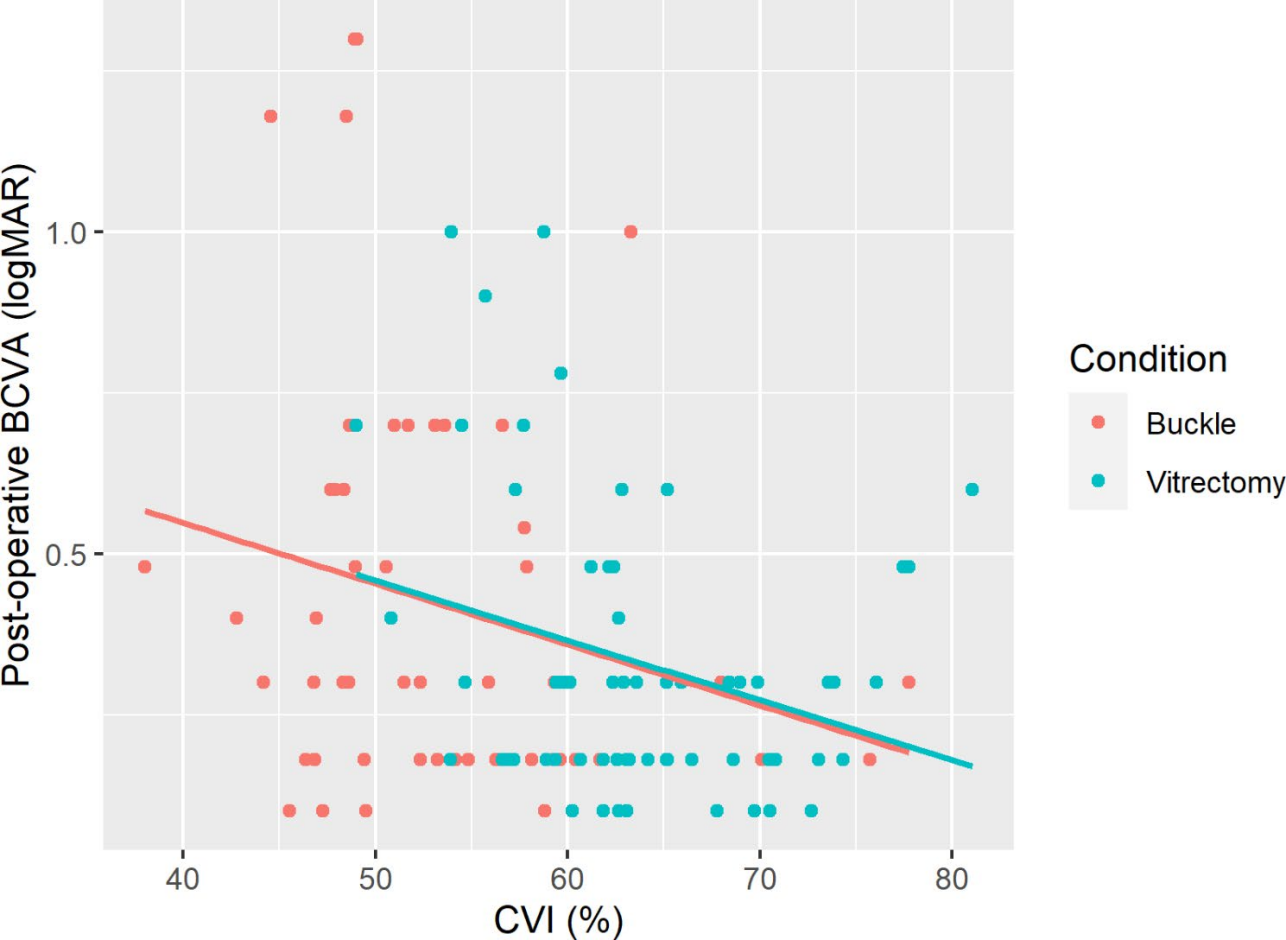
**Negative correlations between the CVI and postoperative BCVA.** The choroidal vascularity index (CVI) and the postoperative BCVA of the surgical cases are plotted and colored according to the surgical treatment. The colored lines represent the linear regression of the data for each surgical treatment condition. The negative slope of the regression lines indicates an inverse relationship between the CVI and postoperative BCVA for both surgical groups

Among the surgical cases, a negative correlation was found between CVI and postoperative BCVA (Pearson correlation = -0.298); that is, eyes with a higher CVI tended to have a lower logMAR (i.e., better vision) and vice versa. This negative correlation was observed in both the vitrectomy and buckling groups (Fig. 2; Pearson’s correlation = -0.277 for the vitrectomy group and − 0.233 for the buckling group).

Next, a multivariate linear model was created to identify the potential relationship or correlation between patient characteristics and postoperative BCVA among surgical cases. For this analysis, the variables in the model included age, CVI, number of weeks with a detached macula, and follow-up time after surgery. Of the four variables in the model, only CVI showed a significant negative correlation with postoperative BCVA (Table 4, *p* = 0.0027, coefficient = -0.0090).

**Table 4:** Multivariate linear regression model of postoperative BCVA.

Variables	Estimate of Coefficient	*P*
Age	-0.00145	0.417
CVI	-0.0090	0.0027
Time with a detached macula	0.0054	0.610
Postoperative follow-up	-0.00089	0.665

CVI, Choroidal vascularity index

**Fig. 3:**
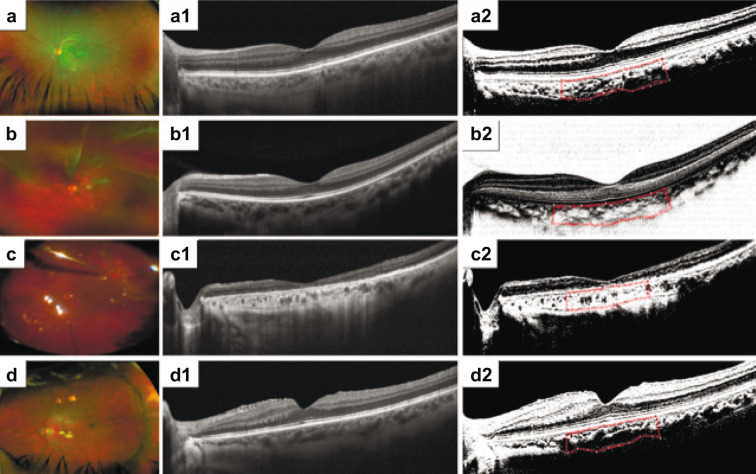
(a) Optos wide-angle photograph of a normal control eye. (a1) Enhanced high-definition (HD) 9-mm horizontal B-scan designed to depict details of the intraretinal structure and subfoveal choroidal layers in a normal eye. (a2) Corresponding horizontal B-scan with binarized processing of the subfoveal choroidal stroma and luminal vascular visualization of the subfoveal choroidal vessels for obtaining the choroidal vascularity index (CVI) of a normal emmetropic eye. The selected subfoveal area is clearly delineated with a red dotted line. (b) Clinical example involving a 59-year-old symptomatic male complaining of an acute decrease in vision in his left eye, progressing over seven days. The preoperative visual acuity was 20/400, and the applanation ocular tension was 10 mmHg. Fundus examination showed a baggy rhegmatogenous macula-off RDD with a solitary arrow-shaped superior retinal tear on M I-II. Retinal surgery was performed by primary vitrectomy. The vitreous base was carefully shaved, and the superior retinal tear was released and marked by endodiathermy. Perfluoro-carbon liquid-assisted endodrainage was performed. The retinal break was treated with an argon endolaser, and fluid-gas exchange was performed using a non-expandable 15% perfluoropropane gas mixture at the end of the procedure. After 8 months of serial follow-up, the eye had a BCVA of 20/40 (logMAR) at the patient’s last visit. (b1) On postoperative enhanced HD 9-mm horizontal B-scan, a normal postoperative foveal profile is depicted with well-defined inner and outer retina layer biomarkers, no residual subretinal fluid (SRF) and well-defined choroidal vessels. (b2) Corresponding horizontal B-scan with binarized processing depicting a normal relationship between the total choroidal area (TCA) and luminal area (LA). The subfoveal binarized area is delineated with a red dotted line. The CVI is equal to that of the fellow eye. (c) Transurgical image of a 71-year-old female who underwent primary vitrectomy in her left eye because of a 12-day history of symptomatic pseudophakic acute rhegmatogenous RD. The preoperative BCVA was 20/200 (logMAR), and her eye was treated with three-port 25-g pars plana vitrectomy (PPV). Fluid-air gas exchange was performed with 15% C_3_F_8_ tamponade. After 18 months of postoperative follow-up, the operated eye showed a best corrected visual acuity of 20/40 (logMAR 0.30). (c1) A long-term postoperative horizontal B-scan through the fovea depicts a normal foveal profile with sclerotic, medium-sized choroidal vessels. (c2) Corresponding horizontal B-scan binarized image of the subfoveal choroidal stroma and luminal vascular visualization of the subfoveal choroidal vessels depicting a lower-than-normal choroidal perfusion index in the fellow eye. (d) Postoperative image of a representative eye from the buckle group. The participant was a 49-year-old male who presented with a 3-day history of progressive metamorphopsia and acute vision loss due to an acute superior-in-origin rhegmatogenous RD due to superior trophic holes inside an area of lattice zone degeneration, with evidence of acute posterior vitreous detachment and a bullous rhegmatogenous macula-off RD. He underwent a 25-g three-port PPV complemented with a 360º scleral buckle on his phakic eye. The preoperative BCVA was 20/200 (logMAR), while that after 7 months of follow-up was 20/60 (logMAR). (d1) Enhanced HD 9-mm image depicts an irregular foveal profile with identifiable inner and outer biomarkers, no presence of residual SRF and evidence of epiretinal membrane proliferation. (d2) Corresponding binarized image. The binarized subfoveal area is delineated with the red dotted line. The CVI was 56.8%, lower than that in the fellow eye

Representative normal controls and surgical participants who underwent enhanced OCT imaging evaluation and CVI calculation are represented in Fig. 3.

## Discussion

In this study, we evaluated the CVI values in 115 eyes with RRD that successfully underwent surgical treatment and 20 healthy control eyes as a comparative control group. Of the 115 surgically treated eyes, 64 underwent PPV and 51 underwent SB combined with vitrectomy. After surgery, the visual outcomes significantly improved in both surgical groups, however, the visual acuity remained inferior to that of the control eyes. Postoperative OCT images were obtained and analyzed to calculate CVI values. Intriguingly, when compared to those in the control eyes, the CVI values were significantly higher in eyes that underwent vitrectomy alone and lower in eyes that underwent both vitrectomy and SB. Additionally, negative correlations were identified between CVI and postoperative BCVA (represented in logMAR units) in both surgical groups when analyzed separately and in the combined surgical sample set. Multivariate linear regression analysis revealed that the CVI was the only one of the four relevant variables that was significantly inversely correlated with postoperative BCVA. Our findings provide important insights into the postoperative choroidal state of eyes with RRD treated with different surgical procedures and the connection between the choroidal vasculature and visual outcomes.

RRD is a vision-threatening condition that can cause permanent blindness without surgical intervention [24]. SB has long been considered the “gold standard” for RRD surgery, yielding a success rate greater than 94% [25]. The postsurgical visual outcomes tend to be good, and the results appear to be long-lasting, with a 95% reattachment rate 20 years after surgery [26]. Some common complications after SB include PVR and choroidal detachment [25]. Additionally, intraoperative hemorrhage may occur, and the procedure is associated with prolonged recovery time, pain, refractive error, floaters, and other ocular disturbances.

In the past, PPV was primarily used in complicated cases of RRD; however, it has become the primary surgical approach in recent years [25]. Some of the advantages of PPV include the ability to visualize all retinal tears and the removal of opacities and ocular synechiae. Additionally, PPV has a high, 95% final anatomic success rate after two surgeries and few intraoperative complications [27]. The disadvantages of vitrectomy include specific positioning and travel restrictions after the surgery. Surgical outcomes can be improved by combining PPV with SB, a combination that has recently been demonstrated to be slightly more favorable in children with giant retinal tear-related detachments [28].

In our study, comparing 64 eyes that underwent PPV alone and 51 eyes that underwent SB combined with PPV, both groups showed substantial visual improvement after surgery. The final postoperative visual acuity was comparable to that in previous studies on surgery in cases with macula-off RRD [29, 30]. We did not observe any significant difference in the final visual outcome between the two surgical groups, which is consistent with a recent study by Ong et al. [28]. However, it is worth pointing out that the p-value of this comparison was trending towards significance with worse visual outcomes in the buckling group compared to the vitrectomy group. Additionally, the postoperative BCVA in eyes with RRD remained significantly worse than that in control eyes, which demonstrates the long-lasting impact of the disease on functional vision. Indeed, multiple OCT angiography studies have identified altered retinal vasculature (foveal avascular zone area, vessel density, and flow density in retinal and choroidal layers) after RRD, which is correlated with visual acuity recovery [31].

The choroid is an important vascular structure that resides underneath the RPE and functions in providing nutrients to the outer retina layers [32, 33]. It plays a key role in maintaining homeostasis and its dysfunction is at the center of many diseases that affect the back of the eye. In vivo, noninvasive observation of the choroid is relevant to both disease diagnosis and treatment but can be challenging because this structure is located beneath multiple layers of tissue. Recent advancements in OCT and the introduction of OCT angiography have substantially improved our ability to visualize the choroid [32]. With these improvements in imaging, it has become increasingly clear that the choroid is much more involved in pathological conditions of the eye.

Better access to visualization of the choroid has resulted in innovations in the quantitative characterization of the choroidal vasculature. CVI is a relatively new biomarker of choroidal vasculature that was first introduced by Agrawal et al. in 2016 [13]. This approach was developed based on the need for a more accurate and robust assessment of the choroidal vasculature system [14]. Since the initial introduction of this metric, the application of CVI has gained popularity, with over 270 citations of the original paper by the second half of 2022. Additionally, CVI has been applied in a diverse array of retinal diseases, such as diabetic choroidopathy, uveitis, central serous chorioretinopathy, retinal vein occlusion, and many other conditions [13, 17, 34–36]. Recent reports have shown that CVI is a promising biomarker for the diagnosis and follow-up evaluation of retinal diseases [37].

In this study, the CVI was calculated for all treatment groups. Consistent with the initial report by Agrawal et al. [13], we found that approximately two-thirds of the subfoveal choroid was vascular in the control emmetropic eyes. The CVI values reported by Agrawal et al. were slightly higher than our measurements (approximately 66% in Agrawal et al. [13] versus 57% in the current study’s control group), perhaps because of the different ethnic backgrounds of the study populations (Malay versus Hispanic subjects).

Intriguingly, significant differences in CVI were identified between the two surgical groups. Specifically, RRD eyes treated with vitrectomy alone were associated with higher CVI values, whereas RRD eyes treated with SB combined with vitrectomy were associated with a lower CVI. The evaluation of CVI in eyes with RRD has been limited, but one study that included 45 eyes with macula-off RRD found that CVI significantly improved 3 months after surgery [38]. However, it remains unclear why the CVI was lower after one surgical procedure, while it was higher after another related procedure. Conceivably, the difference in CVI between the two procedures may be due to the possible side effects of SB on the choroid. A meta-analysis of complications of SB and vitrectomy found that choroidal detachment occurred after the procedure in 3.1% of SB-treated eyes and 0% of vitrectomy-treated eyes [39].

It is also worth noting that it remains unclear why CVI was higher in the vitrectomy group than in the normal control group. The current CVI literature suggests that this metric is variable and can vary depending on the disease [37]. Previous studies have found that CVI is lower in eyes with retinitis pigmentosa [40], diabetic retinopathy [41], glaucoma [42], and geographic atrophy [43]. In contrast, in a series of other diseases, including uveitis [44], central serous chorioretinopathy [35], and neovascular age-related macular degeneration [45], CVI was shown to be higher. These previous studies have suggested that CVI can be influenced by different diseases, possibly due to underlying differences in the pathophysiology and changes in the choroid vasculature. Thus, the variation in CVI among the three different study groups in the present study may be due to both the presence of RRD and the type of therapeutic intervention. More data on both pre-operative and post-operative CVI may shed light on this area.

Given the important role of the choroid in supporting retinal homeostasis, it is worth identifying any potential correlation between the status of the choroidal vasculature and visual acuity. Here, we performed a correlation analysis between the CVI and postoperative BCVA in patients with RRD. In both surgical groups, there was a negative correlation between the two variables. Since visual acuity is measured in logMAR, this result suggests that a higher CVI is correlated with a lower logMAR BCVA and, hence, better vision. Ratra et al. also reported a similar negative correlation between CVI and BCVA in a cohort of 39 patients with Stargardt’s disease [46].

RRD is typically considered a medical emergency, as retinal detachment restricts the blood supply to the neurosensory retina, thus leading to its degeneration. The duration of central vision loss appears to be an important factor in determining postoperative visual acuity [47]. In the present dataset, we included the length of time with a detached macula as one of the variables in the multivariate linear regression analysis of postoperative visual outcomes to investigate the potential relationship between the two variables. We did not find that the time of retinal detachment had a significant influence on the final visual outcomes. Similarly, age and postoperative follow-up time did not significantly influence postoperative BCVA. It is unclear why the length of retinal detachment did not show a significant influence on the visual outcome in this study; perhaps, a larger sample size may be needed.

Of the four variables included in the model, only CVI showed a significant positive effect on visual outcomes. In our dataset, eyes with higher CVI values had lower logMAR values for BCVA, indicating a better final visual ability after surgery. Thus, both correlation and multivariate regression analyses indicated that CVI and postsurgical visual acuity were associated, suggesting that CVI may be a potential biomarker for visual function. Altogether, these data support the notion that a more extensive choroidal vasculature supports the retinal tissue and translates to a better visual outcome in patients with RRD.

Several factors limited the findings of this study. First, it was retrospective in nature and contained a relatively small sample size. Second, only postoperative CVI data were available for analysis in surgically treated eyes. It would be relevant to evaluate the changes in CVI both before and after surgery to identify the influence of RRD and different surgical procedures on choroidal vasculature. Despite these limitations, we performed a novel analysis of the long-term effects of RRD surgery on the choroid, using CVI as a biomarker. Additionally, we analyzed the corresponding visual acuity data, which demonstrated a correlation between choroidal vasculature and vision. Furthermore, we evaluated two surgical procedures, revealing insights into the effects of SB on the choroid vasculature. Although our results are illuminating, further confirmatory studies should be performed to verify these findings.

## Conclusion

RRD is a vision-threatening disease that requires urgent surgical intervention to preserve and restore vision. The SB procedure has long been successfully used for the surgical repair of RRD, and vitrectomy has recently become popular. In a dataset consisting of 115 eyes with RRD that underwent successful surgery and 20 control eyes, we found that visual acuity significantly improved after surgery but was still inferior compared to that of healthy eyes. CVI is a relatively new metric of the choroid vasculature, an important issue for maintaining retinal homeostasis. The long-term postoperative CVI was elevated in eyes treated with vitrectomy alone and reduced in eyes treated with vitrectomy and SB. In both treatment groups, there was a significant positive association between CVI and functional vision, highlighting the role of the choroid in supporting vision. The present investigation provides important insights into the long-term functional and choroidal outcomes after the two types of RRD surgery, which should be further validated by additional studies.

## Electronic supplementary material

Below is the link to the electronic supplementary material.

Supplementary Material 1

## Data Availability

The datasets used in this study are included in the main article. Photographs and figures from this study may be released via a written application to the Photographic Laboratory and Clinical Archives Department of the Retina Specialists Unit at Oftalmologia Integral ABC, Medical and Surgical Assistance Institution (nonprofit organization), Av. Paseo de las Palmas 735 suite 303, Lomas de Chapultepec, Mexico City 11,000, Mexico and the corresponding author upon request. All the analysis files and figures (pdf, eps, tiff) can be found at the following link: https://www.dropbox.com/sh/ixevyamp2r0188h/AADERi0TSj2If0yeFYejs4l5a?dl=0.

## Abbreviations

BCVA: best corrected visual acuity
CVI: choroidal vascularity index
LA: luminal area
LogMAR: logarithm of the minimum angle of resolution
MTM: myopic traction maculopathy
OCT: optical coherence tomography
PPV: pars plana vitrectomy
PVR: proliferative vitreoretinopathy
RPE: retinal pigment epithelium
RRD: rhegmatogenous retinal detachment
SD-OCT: spectral-domain OCT
SRF: subretinal fluid
TCA: total choroidal area
VEGF: Vascular endothelial growth factor
