# Correction: Postoperative choroidal vascularity index after the management of macula-off rhegmatogenous retinal detachment

**DOI:** 10.1186/s40942-023-00464-x

**Published:** 2023-04-11

**Authors:** Miguel A. Quiroz-Reyes, Erick A. Quiroz-Gonzalez, Miguel A. Quiroz-Gonzalez, Virgilio Lima-Gomez

**Affiliations:** 1 Oftalmologia Integral ABC, Retina Department, Medical and Surgical Assistance Institution (Nonprofit Organization), Affiliated with the Postgraduate Studies Division, National Autonomous University of Mexico, Av. Paseo de las Palmas 735 Suite 303, Lomas de Chapultepec, 11000 Mexico City, Mexico; 2 Retina Department, Oftalmologia Integral ABC, Medical and Surgical Assistance Institution (NonprofitOrganization), National Autonomous University of Mexico, Mexico City, Mexico; 3 Institute of Ophthalmology, Fundacion Conde de Valenciana, Medical and Surgical Assistance Institution (Nonprofit Organization), Affiliated with the Postgraduate Studies Division, National Autonomous University of Mexico, Av. Paseo, Mexico City, Mexico; 4 Juarez Hospital, Public Assistance Institution (Nonprofit Organization), Av. Politecnico Nacional 5160, Colonia Magdalena de las Salinas, 07760 Mexico City, Mexico

**Correction: International Journal of Retina and Vitreous (2023) 9:19** 10.1186/s40942-023-00454-z

Following publication of the original article [1], the authors identified an error in their article. Figure 3 was erroneously omitted from the published article. Figure 3 is given in this correction article. The original article [1] is corrected.

**Fig. 3:**
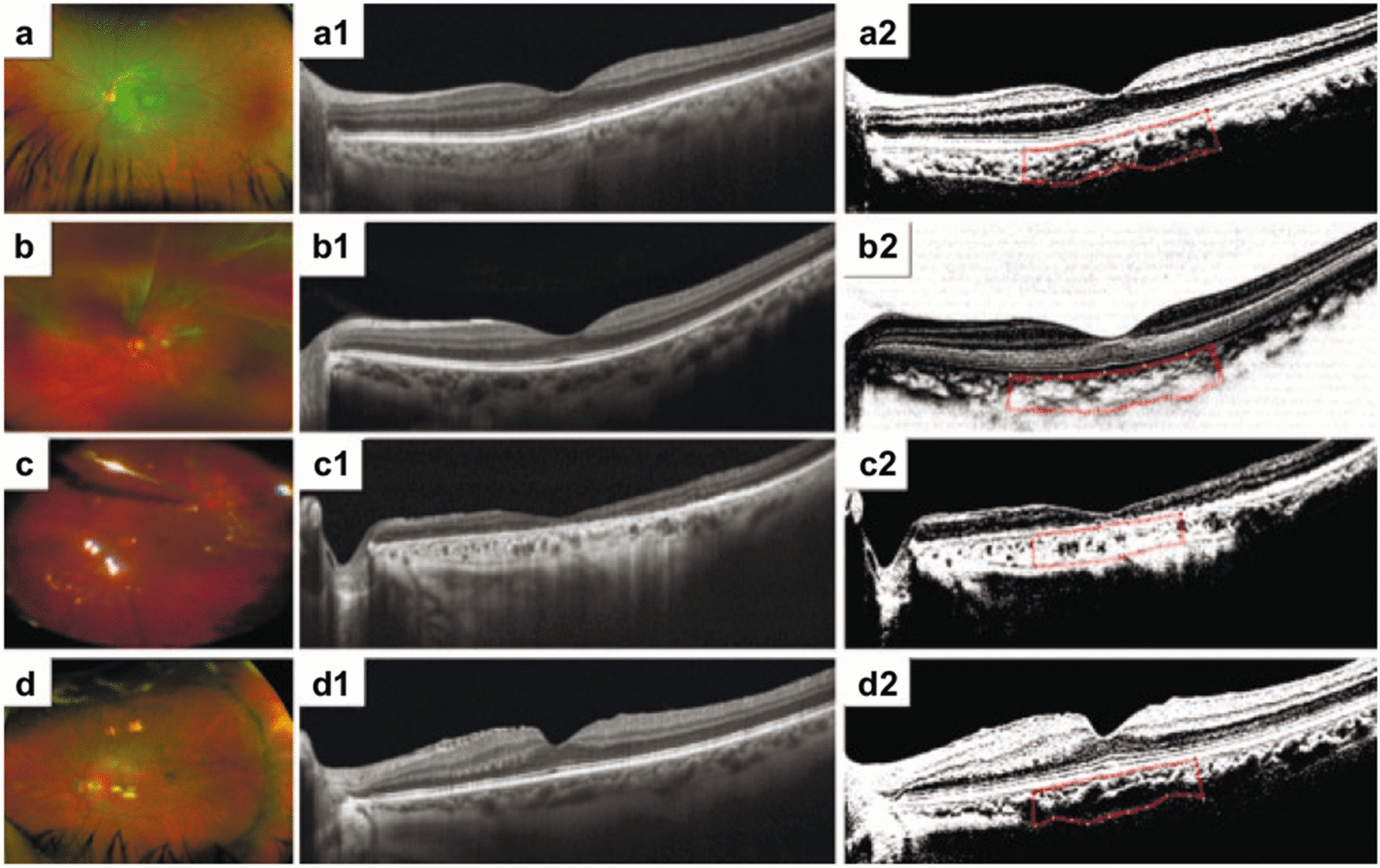
**a** Optos wide-angle photograph of a normal control eye. **a1** Enhanced high-definition (HD) 9-mm horizontal B-scan designed to depict details of the intraretinal structure and subfoveal choroidal layers in a normal eye. **a2** Corresponding horizontal B-scan with binarized processing of the subfoveal choroidal stroma and luminal vascular visualization of the subfoveal choroidal vessels for obtaining the choroidal vascularity index (CVI) of a normal emmetropic eye. The selected subfoveal area is clearly delineated with a red dotted line. **b** Clinical example involving a 59-year-old symptomatic male complaining of an acute decrease in vision in his left eye, progressing over seven days. The preoperative visual acuity was 20/400, and the applanation ocular tension was 10 mmHg. Fundus examination showed a baggy rhegmatogenous macula-off RDD with a solitary arrow-shaped superior retinal tear on M I-II. Retinal surgery was performed by primary vitrectomy. The vitreous base was carefully shaved, and the superior retinal tear was released and marked by endodiathermy. Perfluoro-carbon liquid-assisted endodrainage was performed. The retinal break was treated with an argon endolaser, and fluid-gas exchange was performed using a non-expandable 15% perfluoropropane gas mixture at the end of the procedure. After 8 months of serial follow-up, the eye had a BCVA of 20/40 (logMAR) at the patient’s last visit. **b1** On postoperative enhanced HD 9-mm horizontal B-scan, a normal postoperative foveal profile is depicted with well-defined inner and outer retina layer biomarkers, no residual subretinal fluid (SRF) and well-defined choroidal vessels. **b2** Corresponding horizontal B-scan with binarized processing depicting a normal relationship between the total choroidal area (TCA) and luminal area (LA). The subfoveal binarized area is delineated with a red dotted line. The CVI is equal to that of the fellow eye. **c** Transurgical image of a 71-year-old female who underwent primary vitrectomy in her left eye because of a 12-day history of symptomatic pseudophakic acute rhegmatogenous RD. The preoperative BCVA was 20/200 (logMAR), and her eye was treated with three-port 25-g pars plana vitrectomy (PPV). Fluid-air gas exchange was performed with 15% C_3_F_8_ tamponade. After 18 months of postoperative follow-up, the operated eye showed a best corrected visual acuity of 20/40 (logMAR 0.30). **c1** A long-term postoperative horizontal B-scan through the fovea depicts a normal foveal profile with sclerotic, medium-sized choroidal vessels. **c2** Corresponding horizontal B-scan binarized image of the subfoveal choroidal stroma and luminal vascular visualization of the subfoveal choroidal vessels depicting a lower-than-normal choroidal perfusion index in the fellow eye. **d** Postoperative image of a representative eye from the buckle group. The participant was a 49-year-old male who presented with a 3-day history of progressive metamorphopsia and acute vision loss due to an acute superior-in-origin rhegmatogenous RD due to superior trophic holes inside an area of lattice zone degeneration, with evidence of acute posterior vitreous detachment and a bullous rhegmatogenous macula-off RD. He underwent a 25-g three-port PPV complemented with a 360º scleral buckle on his phakic eye. The preoperative BCVA was 20/200 (logMAR), while that after 7 months of follow-up was 20/60 (logMAR). **d1** Enhanced HD 9-mm image depicts an irregular foveal profile with identifiable inner and outer biomarkers, no presence of residual SRF and evidence of epiretinal membrane proliferation. **d2** Corresponding binarized image. The binarized subfoveal area is delineated with the red dotted line. The CVI was 56.8%, lower than that in the fellow eye
