# Supplementary material for: Postoperative choroidal vascularity index after the management of macula-off rhegmatogenous retinal detachment
Source: Int J Retina Vitreous. 2023 Mar 29;9:19. doi: 10.1186/s40942-023-00454-z (PMC10053660; doi:10.1186/s40942-023-00454-z)
Supplement: Supplementary file 1 — Supplementary Material 1 [file 40942_2023_454_MOESM1_ESM.docx]

**Response to Reviewers**

Reviewer #1: The introduction is so long .I suppose it is to be cut to half. There is so about the surgical procedure .

Please go to the point	"CVI ".

Last part in introduction is about methods and need to be cut.

Author response: The entire introduction section has been significantly shortened to improve clarity and the description of surgical procedures has been reduced.

Authors believe that higher CVI is correlated with better vision .

When comparing two surgical groups there is no significant difference between groups in visual function but significant difference exists in CVI between groups. Please explain more.

What is your idea about higher CVI in vasectomy group that normal control group?

Please explain

Author response: Correlation analysis found higher CVI is correlated with lower logMAR (better visual function) in both surgical groups, separately and combined. While data on correlation of CVI to BCVA is limited in the literature, we added in the discussion section a reference to Ratra et al. (2018) that reached the same conclusion in a cohort of Stargardt disease patients: “Ratra et al. also reported a similar...”

The reviewer made the excellent point that no significant difference was detected between the two groups in visual function, but a significant difference was detected in CVI. For BCVA, the p-value of post-operative BCVA was trending towards significance (*p* = 0.168) with the Buckling group having higher logMAR than the Vitrectomy group. This is consistent with the CVI data that showed lower CVI in the Buckling group than the Vitrectomy group (*P* < 0.0001). We have added additional emphasis of this observation in the discussion section: “We did not observe any statistically significant difference in...”

Regarding the higher CVI in Vitrectomy group compared to control group, it remains unclear and may require additional CVI data from pre-surgical RRD eyes. We also noted, for certain diseases, CVI appeared to be elevated and for others reduced. Thus, CVI in the current study eyes is influenced by both the disease and surgical treatment. We have revised the discussion section to highlight these points: “It is also worth noting it remains unclear why CVI was higher...”

Reviewer #2: Review of the article "POSTOPERATIVE CHOROIDAL VASCULARITY INDEX AFTER THE MANAGEMENT OF MACULA-OFF RHEGMATOGENOUS RETINAL DETACHMENT - IJRV-D-23-00006."

The article aims to compare the choroidal vascularity index in retinal detachment eyes between patients treated with vitrectomy and patients treated with vitrectomy and buckling. The study has a critical bias in sample selection and problematic study design. I suggest restructuring the study and the analyses.

Major comments

Background

Scleral buckling is the surgical technique described on page 5, lines 106-110; however, in the study case, the scleral buckling was associated with vitrectomy.

Scleral buckling associated with vitrectomy has a different physical principle, pathophysiology, and surgical criteria. I suggest rewriting the paragraphs for scleral buckling and changing for scleral buckling associated with vitrectomy.

Author response: we are sorry for the confusing descriptions. We have revised the introduction to improve clarity and to briefly describe the current literature on the combined scleral buckling with vitrectomy approach. We also added references to two recent review articles that covered each procedure and the combined approach in detail: “Scleral buckling is a surgical technique that repairs...”

Methods

I suggest reviewing the study design. A retrospective study by design is prospective.

Author response: Thank you for your recommendation and encouraging comments. You are absolutely right. The study design was intended to reduce confounding factors by retrospectively analyzed only primary, non-complicated RRDs treated with two surgical approaches. Due to retrospective analysis, a nonrandomly selected group of patients with non-complicated primary RRD who underwent primary Vitrectomy vs scleral buckling combined with vitrectomy were included. The rate and type of complication were not assessed in this study. Only, the postop CVI was measured in both groups to evaluate the role of encircling elements on the choroidal circulation and its potential repercussion on the final BCVA.

There is a critical bias in the selection criteria. The surgical criteria for choosing vitrectomy or vitrectomy and scleral buckling are not the same, and therefore comparing the groups are challenging.

Author response: Absolutely agreed with you. However, in our current surgical practice we tend to treat the uncomplicated RRD with vitrectomy in pseudophakic, multiple or posterior breaks, vitreous opacities, to avoid scleral buckle-related complications and the potential deleterious repercussion on the choroidal circulation. In this study, only more complicated cases were non-randomly selected to undergo scleral buckling combined with vitrectomy according to surgeons’ preferences. A supplementary buckling is added in case of primary RRD with inferior breaks or in cases where breaks are not found in spite. In this way, the study aimed to comparatively analyze the effect of scleral buckling combined with vitrectomy vs vitrectomy alone on the choroidal vascularity index (CVI) and correlate it with BCVA. Unfortunately, the sample size is too small to standardize surgical trends.

Does the OCT (Spectralis or RTVue) have an exam protocol? For comparison purposes, I suggest standardizing the time span and keeping the same OCT equipment. Inter-machine OCT variations may be a confounding factor in the analysis.

Author response: The OCT exam protocol used in our current practice is to do postoperative OCT evaluation as soon as the gas bubble is reduced enough to allow the beam to take a horizontal B-scan through the macula. Due to the retrospective nature, only patients who showed no presence of submacular residual SRF were evaluated at final follow up visit. Only the postoperative HD images taken with a SD-OCT (RTVue-XR platform SD-OCT; Optovue, Inc., Fremont, CA, USA) were binarized to get the CVI.

In the surgical technique, I suggest reviewing and clarifying the included patients. Does this study include only vitrectomy and vitrectomy + scleral buckling or also include scleral buckling/sponge with late vitrectomy?

Author response: Sorry for the confusion in our narrative. Two study groups as follow: Control group and surgical group (Vitrectomy alone treated group and scleral buckling combined with vitrectomy treated group).

Results

I suggest improving table 1 by adding the patient sex, race, comorbidities, retinal detachment characteristics, number of patients in the control group, and new statistical analyses.

Author response: The results section has been updated to include the data that all eyes are from subjects of Hispanic ethnicity: “The present study included 20 control...” Patient sex, number of patient in each group, and additional statistical results are now included in Table 1. Comorbidities and specific retinal detachment characteristic data are not available in this dataset.

It's not clear the objective of the multivariate analysis in the postoperative BCVA. I suggest reviewing the multivariate logistic regression variables and outcomes.

Author response: the methods and results sections have been revised to clarify that we created a multivariate linear model to “identify potential relationship or correlation between patient characteristics and postoperative BCVA among the surgical cases.” The main findings of the linear model are included in Table 4. The discussion section has been revised to clarify the interpretation of the regression analysis: “In the present dataset, we included the length of time...”

Reviewer #3: - It is important to clarify the amount of cases that underwent multiple surgeries.

Author response: The result has been adjusted to include information on number of additional surgeries: Four of the 64 (6.3%) vitrectomy group cases required additional vitrectomy revision surgery due to redetachment, while 2 of the 51 (3.9%) buckling group cases required additional intervention. The rate and type of complication were not statistically analyzed.

- Are the cases that underwent multiple surgeries the ones associated with lowest BCVA and changes in CVI?

Author response: Due to the small number of cases that required additional surgery, statistical tests are not appropriate for comparison of BCVA and CVI outcomes between eyes that either received or did not receive additional surgery.

Reviewer #4: Abstract: Ok.

Summary statement and Key messages: Ok.

Background

Reference 1: In according with the written text.

Reference 2: In according with the written text.

Reference 3: In according with the written text.

Reference 4: Statement is correct, but I didn't find this mention in the cited study.

Author response: Reference to the sentence “but show better visual acuity outcome when ...” in the introduction has been changed to a large meta-study that found better visual outcome with more immediate intervention. Sothivannan A, Eshtiaghi A, Dhoot AS, Popovic MM, Garg SJ, Kertes PJ, Muni RH. Impact of the Time to Surgery on Visual Outcomes for Rhegmatogenous Retinal Detachment Repair: A Meta-Analysis. Am J Ophthalmol. 2022;244:19-29.

Reference 5: In according with the written text.

Reference 6: Statement is correct, but I didn't find this mention in the cited study ("When left untreated, RRD typically...").

Author response: Reference to the sentence “Without treatment, RRD can lead to ...” in the introduction has been changed to a more suitable study. Iwase T, Kobayashi M, Yamamoto K, Yanagida K, Ra E, Terasaki H. Changes in Blood Flow on Optic Nerve Head After Vitrectomy for Rhegmatogenous Retinal Detachment. Invest Ophthalmol Vis Sci. 2016 Nov 1;57(14):6223-6233.

Reference- 7: In according with the written text.

Reference 8-9: In according with the written text.

Reference- 10: In according with the written text.

Reference- 11: In according with the written text.

Reference- 12: In according with the written text.

Reference- 13: In according with the written text.

Reference- 14: In according with the written text.

Reference- 15-19: In according with the written text.

Methods

Study design: Ok.

Examinations

Reference 20: In according with the written text.

Surgical technique

Reference 21: In according with the written text.

CVI measurement technique: Ok.

Statistical analysis: Ok.

Results

Characteristics of the eyes: Ok.

RRD surgical outcomes: Ok.

CVI analysis:

The images of figures 1 and 2 are in the text, and of figure 3 is at the end of the article. Please review page 14.

Author response: Figure 3 containing figures and images of controls and surgical cases will be place accordingly.

Discussion:

Reference- 22: In according with the written text.

Reference- 23: In according with the written text.

Reference- 24: This success rate was for patients with two interventions. Patients with only 1 intervention the rate was lower, around 70%.

Author response: The associated text in discussion has been revised to: “Additionally, PPV has a high, 95% final anatomic success rate after two operations and few intraoperative complications.”

Reference- 25: In according with the written text.

Reference- 26-27: In according with the written text.

Reference- 28: In according with the written text.

Reference- 29-30: In according with the written text.

Reference- 31-33: In according with the written text.

Reference- 34: In according with the written text.

Reference- 35: In according with the written text.

Reference 37-42: In according with the written text.

Reference 43: In according with the written text.

Conclusion: Ok.

References: Ok.

Figures: Only figures 1 and 2 are identified, the other images are unidentified and are repeated.

Author response: Figure 3 containing figures and images of controls and surgical cases will be place accordingly.

Suggestions to the authors: As visual acuity was similar between patients operated with PPV and PPV+Buckle, but there was a difference in the CVI, other macular functions could be tested, such as: contrast sensitivity, color test and microperimetry.

Author response: We want to thank the reviewer for this thoughtful suggestion. We agree that the mentioned techniques could provide additional valuable insights, but due to the retrospective nature of this study, we do not have access to more datasets.

Thank you for your kind evaluation and consideration.

Miguel A. Quiroz Reyes

Oftalmologia Integral ABC, Retina Specialists Unit (Retina department). Medical and Surgical Assistance Institution (Nonprofit organization) Affiliated with the Postgraduate Studies Division at the National Autonomous University of Mexico, Av. Paseo de las Palmas 735 Suite 303, Lomas de Chapultepec, Mexico City 11000, Mexico
